# Symptoms of Anxiety and Depression in Students With Developmental Disabilities During COVID-19 Lockdown in Poland

**DOI:** 10.3389/fpsyt.2021.576867

**Published:** 2021-03-18

**Authors:** Michal Gacek, Lukasz Krzywoszanski

**Affiliations:** Faculty of Pedagogy and Psychology, Institute of Psychology, Pedagogical University of Krakow, Krakow, Poland

**Keywords:** COVID-19, developmental disabilities, intellectual disability, anxiety, depression, lockdown

## Abstract

**Background:** In this study we aimed to assess symptoms of anxiety and depression in persons with developmental disabilities during COVID-19 lockdown.

**Method:** Soon after school closures related to the pandemic, we conducted telephone interviews with 64 vocational school students with developmental disabilities, the majority of whom had mild intellectual disability, and their parents. The parents were asked about stressful events experienced during lockdown. The students were assessed with screening measures for anxiety (GAD-7) and depression (PHQ-8).

**Results:** Over one third of the tested students reported mild or more severe symptoms of anxiety and depression, and girls were more affected than boys. The number of experienced lockdown inconveniences predicted the severity of depression symptoms in girls.

**Discussion:** The high prevalence of symptoms of anxiety and depression in persons with developmental disabilities indicates the need for screening studies and the provision of psychological help in situations such as the COVID-19 lockdown.

## Introduction

Since the new coronavirus disease (COVID-19) was first identified in Wuhan, China, in December 2019 (1) it has rapidly spread to other regions of China and other countries, thus becoming a major concern for global public health (2). The first cases of COVID-19 in Europe were reported in February in Italy, and this country is one of the most seriously affected by the disease in the region (3). The first case of COVID-19 in Poland was identified at the beginning of March 2020 (4), a week before the WHO's director-general declared a state of pandemic (5). The precautions officially introduced in March by the Polish government included closures of schools and universities, closures and limitations in trade and services, and significant movement restrictions for citizens (6). After the 1st of April, persons under the age of 18 could leave home only in the presence of a parent or caregiver. The restrictions also concerned persons with developmental disabilities and their families. However, in the context of the problems that affected everyone in the country, little attention seemed to be paid to the situation of this particular group.

The study presented in this article aimed to investigate the symptoms of anxiety and depression in vocational school students with developmental disabilities who stayed home during the pandemic situation due to school closures. The studied group consisted of persons with developmental disabilities, the majority of whom had intellectual disabilities; this is a vulnerable group whose risk of psychopathology is ~4–5 times higher than in the general population (7), and three to four times higher for persons with intellectual disability (8). The higher prevalence of mental health issues also applies to symptoms of anxiety and depression (9). The sex differences in general population indicate that women are at significantly greater risk of anxiety and depression than men (10). Findings related to persons with ID also suggest a higher prevalence of depression in girls (11), and that girls with ID are significantly more fearful and experience more worry than boys (12).We assumed that the burden related to the pandemic situation may exacerbate emotional difficulties in this group. Similar assumptions in regard to general population were made and confirmed in a study conducted in Ireland during the pandemic (13). The study allowed us to obtain information about these difficulties and to identify persons with developmental disabilities in need of psychological or psychiatric help.

## Method

### Ethical Considerations

The study was approved by the ethical committee at the Pedagogical University of Krakow and by the school board of the special education center in which it was conducted. During school enrolment, the parents or primary caregivers of all the participants in the survey gave their written consent to receive an invitation to participate in scientific research. Detailed information about this study was given during telephone conversations and consent was obtained orally from the participants. The participants were informed that they could withdraw from the study at any time. The data was kept and used in accordance with the General Data Protection and Regulation Law in force (14).

### Participants

We assessed 64 students (30 boys) from a vocational school for persons with developmental disabilities (M_*age*_ = 18.5 years; SD = 2.25) along with their parents or primary caregivers. Included in our study were 62 students with a diagnosis of mild intellectual disability, seven persons with a diagnosis of intellectual disability comorbid with a physical disability, and one with visual impairment. Two students had a diagnosis of an autism spectrum disorder. In each case, the diagnoses were provided by a psychological-pedagogical counseling center. The mild intellectual disability was diagnosed based on the ICD-10 (15) criteria. The results of a priori analysis of statistical power for two independent Pearson's coefficients with effect size defined as *q* = 0.75 showed that for error probability set as α = 0,05 and power set as 1-β = 0.8 the minimum required sample size was 62, when equal sizes of both subgroups were assumed.

### Measures

#### Interview Regarding the Lockdown Situation

The interview comprised seven questions that required a “yes” or “no” answer about difficulties that persons experienced during the last 2 weeks of COVID-19 lockdown. The parents or primary caregivers were asked how often they had sought information regarding the outbreak, whether they had experienced trouble accessing the internet or buying basic food products, hygiene products, or regularly used medications, as well as if they had any difficulties in dealing with official matters. The participants were also asked if anyone at home during the last 2 weeks had lost employment or the opportunity to do their job, and if anyone had felt ill or experienced a substantial worsening of their medical condition.

#### Generalized Anxiety Disorder 7

Generalized Anxiety Disorder 7 (GAD-7) is a brief questionnaire developed for screening purposes by Spitzer et al. (16). This tool is intended to measure the severity of symptoms of anxiety. We used a Polish translation of the questionnaire available on the Patient Health Questionnaire website (17). This measure has been linguistically validated by the MAPI Research Institute (18) in accordance with the international standards of cultural adaptation methodology described in the Linguistic Validation Manual for Health Outcome Assessments (19) and ISPOR guidelines for the cross-cultural adaptation (20).

#### Patient Health Questionnaire – 8

Patient Health Questionnaire – 8 (PHQ-8) is a brief questionnaire based on the widely used PHQ-9 screening measure for depressive symptoms (21). The only difference between PHQ-8 and PHQ-9 is that the former questionnaire does not include the last question regarding suicidal ideation. The PHQ-8 has been suggested as a better measure than PHQ-9 since the item regarding suicidal ideation is considered inaccurate (22). Also, the PHQ-8 is suggested as a screening measure when a study is conducted *via* telephone due to ethical reasons (23). The items in the Polish version of the questionnaire have been linguistically validated (18) and were obtained from the official site (17).

### Procedure

The study was conducted in Krakow in March and April 2020, after school closures due to the pandemic outbreak. We obtained the approval to conduct this research from the school board of a special education center for adolescents and young adults with intellectual disabilities. The data was gathered by two school psychologists who contacted the students and their parents or primary caregivers by phone. Before the pandemic, the psychologists were familiar with all the students they contacted. After giving consent, the parent or primary caregiver answered the questions regarding inconveniences experienced during lockdown, and the students answered questions included in GAD-7 and PHQ-8. When the participants reported that they had experienced symptoms of anxiety or depression, the psychologists conducting the interview offered them counseling and help in setting up a psychiatric consultation.

### Statistical Analyses

Due to the skewness of the distributions of the total scores in GAD-7 and PHQ-8, the Mann-Whitney *U*-test was used to examine the gender differences in the total scores in both scales with the rank-biserial correlation as a measure of the effect size. The moderated regression analyses based on the ordinary least squares linear model were performed to verify whether the impact of the pandemic-related inconveniencies on the total GAD-7 and PHQ-8 scores differed between girls and boys. The sum of these inconveniences, gender, and their interaction were specified as the predictors in the regression models. The dependent variables were transformed by taking the square root of their initial value to avoid the skewness of the distribution of the residuals in the regression models.

The non-parametric tests and regression analyses were computed using the free and open-source statistical platform jamovi (24), based on the R programming language for statistical computing (25) with the GAMLj jamovi module (26). The plots were prepared using the sjPlot R package (27).

## Results

**Figure 1:**
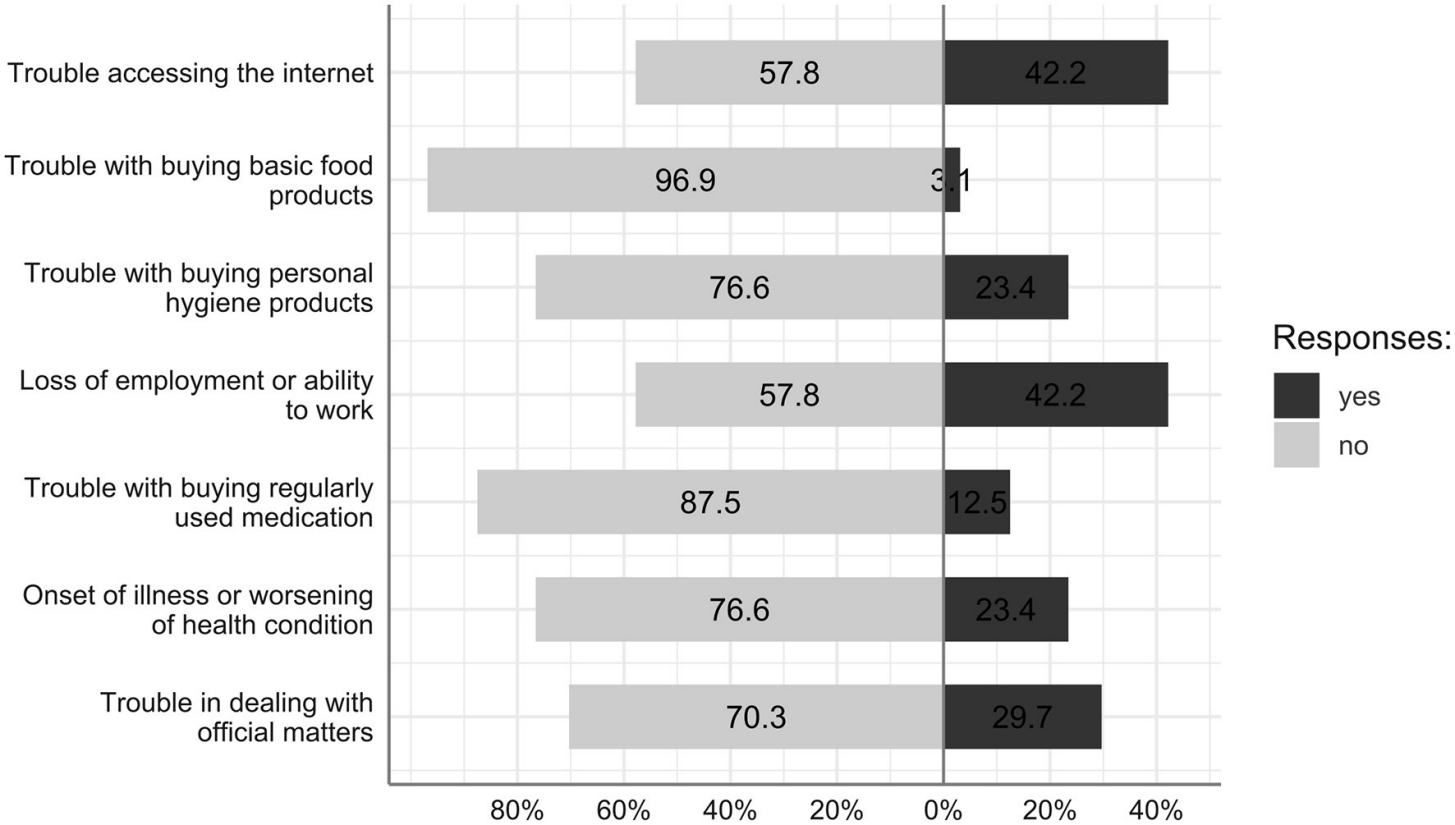
Percentages of responses to the questions about the pandemic-related inconveniencies experienced by anyone in the households of the respondents.

The frequencies for the pandemic-related inconveniencies experienced by anyone in the subjects' households are presented in Figure 1 and additional descriptive statistics are presented in Table 1. Trouble accessing the internet (42.2%), loss of employment or ability to work (42.2%), and trouble in dealing with official matters (29.2%) were the inconveniences most frequently reported by the respondents.

**Table 1:** Descriptive statistics for age, GAD-7, PHQ-8, and sum of reported inconveniences.

**Variable**	**Gender**	**Mean**	**Median**	**Std. Deviation**	**Minimum**	**Maximum**
Age	Boys	18.60	18.5	2.39	16	23
	Girls	18.38	18.0	2.16	15	23
GAD-7 raw scores [sum of points]	Boys	2.33	1	3.51	0	14
	Girls	5.15	3	5.25	0	19
PHQ-8 raw scores [sum of points]	Boys	1.80	0	3.76	0	18
	Girls	3.91	2	4.75	0	16
Sum of reported inconveniences	Boys	2.17	2	1.62	0	5
	Girls	2.15	2	1.91	0	7

**Figure 2:**
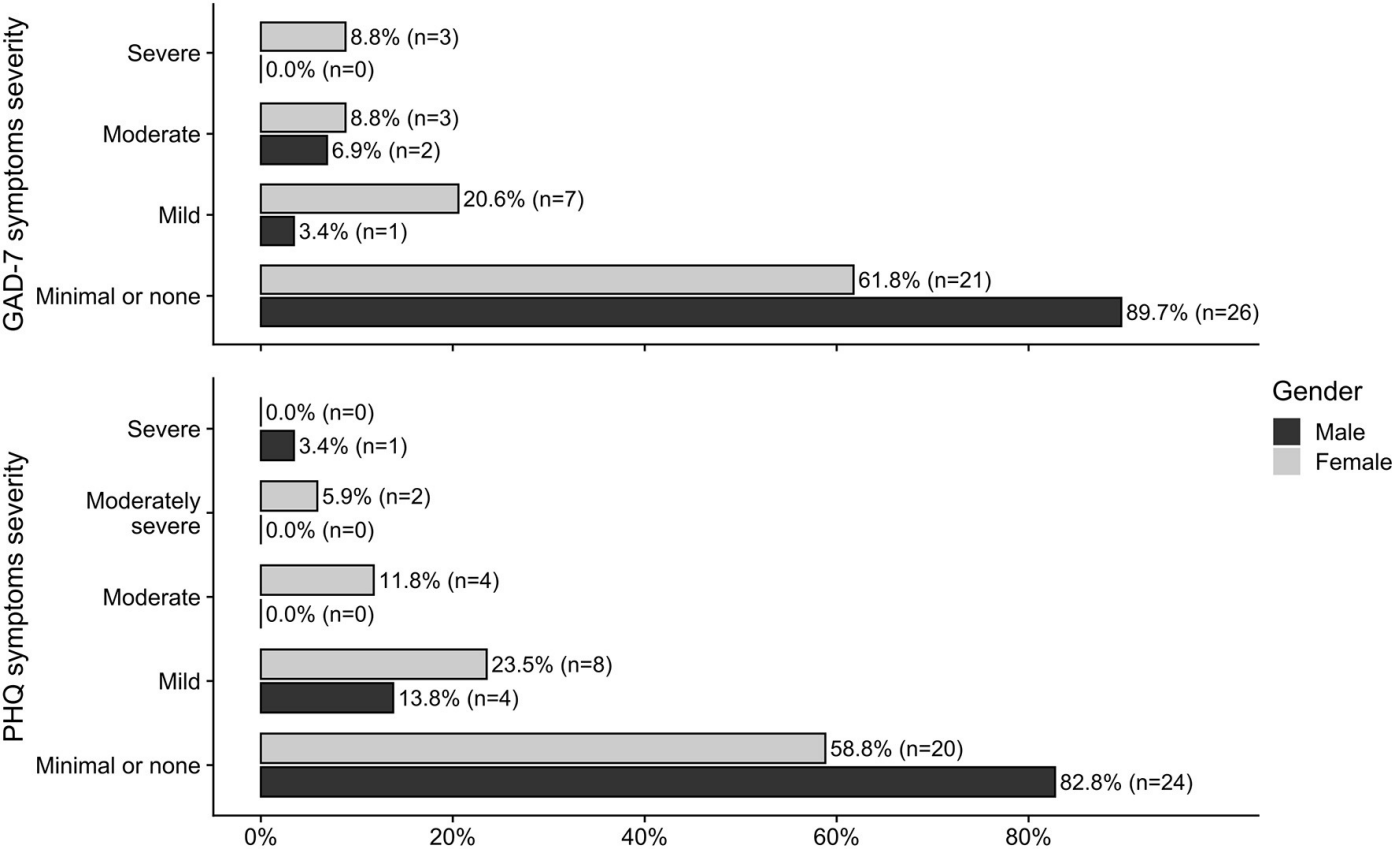
Counts and within-group percentages for females and males classified in diagnostic categories of degrees of the severity of symptoms measured by GAD-7 and PHQ-8.

The results of the Mann-Whitney U test showed that the total scores in GAD-7 were higher in girls than in boys, *U* = 345, *p* = 0.024, *r*_*rb*_ = 0.324, with 95% confidence intervals from 0.050 to 0.552. Girls also obtained higher total scores in PHQ-8 than boys, *U* = 359, *p* = 0.031, *r*_*rb*_ = 0.296 with 95% confidence intervals from 0.020 to 0.531. The raw total scores in GAD-7 and PHQ-8 were re-coded according to the criteria specified in the diagnostic manual (17) into categories representing degrees of symptom severity. The sample distribution for each of the categories is depicted in Figure 2 for boys and girls separately.

**Figure 3:**
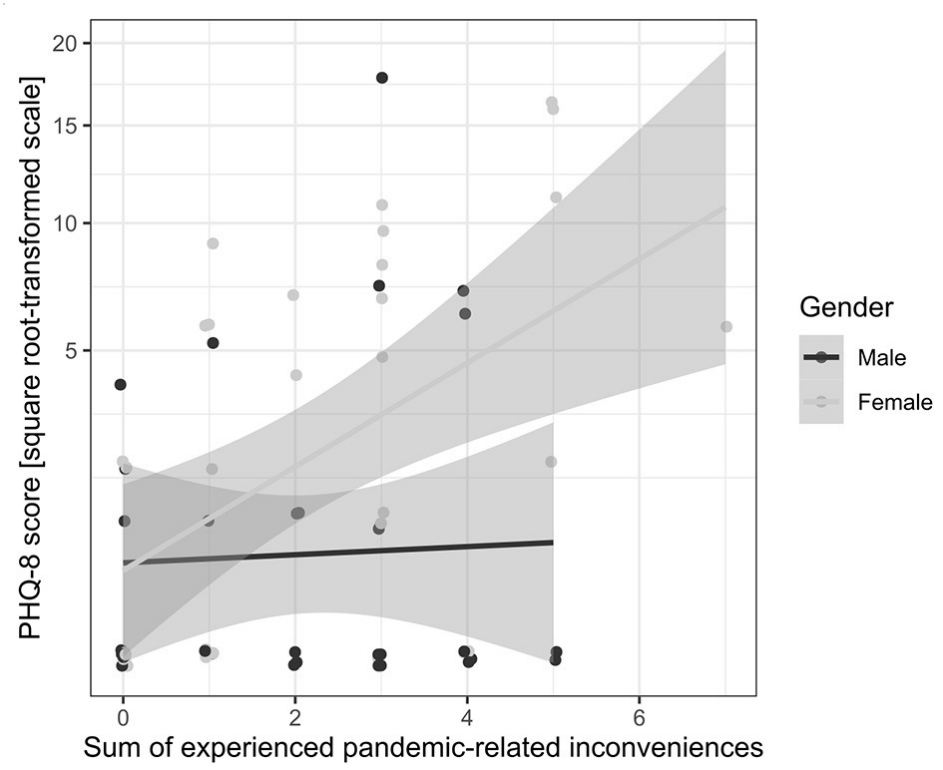
The raw total scores in PHQ-8 plotted against the sum of the pandemic-related inconveniencies experienced by anyone in the household of the respondent, with regression lines fitted separately for females and males. Gray bands show 95% confidence intervals for regression lines.

The overall model *F* test for the multiple regression conducted to predict the square root-transformed GAD-7 total scores from gender, sum of the experienced pandemic-related inconveniencies and their interaction was insignificant, *F*_(3, 60)_ = 2.48, *p* = 0.070, *R*^2^ = 0.110, $R_{adj}^{2}$ = 0.066. The overall model F test for the linear model that predicts the square root transformed PHQ-8 total scores from gender, sum of the experienced pandemic-related inconveniencies and the interaction of both was significant, *F*_(3, 60)_ = 6.09, *p* = 0.001, *R*^2^ = 0.223, $R_{adj}^{2}$ = 0.195. The results of the omnibus tests revealed that the interaction of gender with the sum of the experienced pandemic-related inconveniencies was the only significant effect, *F*_(1, 60)_ = 4.16, *p* = 0.046, *R*^2^ = 0.109, $\eta_{p}^{2}$ = 0.065. This indicates that gender moderates the impact on the total PHQ-8 scores of experienced inconveniencies. As can be inferred from the regression lines and confidence intervals bands shown in Figure 3, in boys the level of predicted total PHQ scores are similar for varying values of the sum of experienced inconveniencies, *b* = 0.029, with CI_95%_ from −0.238 to 0.297, *t*_(28)_ = 0.220, *p* = 0.826, whereas in girls as the sum of the experienced inconveniences increases, the predicted total PHQ scores gradually increase, *b* = 0.378, with CI_95%_ from 0.165 to 0.590, *t*_(32)_ = 3.55, *p* < 0.001.

## Discussion

In our study, we aimed to investigate the symptoms of anxiety and depression during the COVID-19 lockdown in students with developmental disabilities, the majority of whom have been diagnosed with mild intellectual disability. We also interviewed the students' parents or caregivers about the difficulties experienced by the members of their household in the last two weeks. The most-often reported difficulties included trouble with accessing the internet and a household member's loss of employment or opportunity to work. However, it is also worth noting that almost one-fourth of participants' household members reported they experienced the onset of an illness or worsening of their health condition. These results indicate the strong negative influence of COVID-19 lockdown on the health of family members and the financial situation of households of persons with developmental disabilities.

The severity of symptoms of anxiety and depression was higher in girls than in boys with developmental disabilities, which is consistent with results from previous studies (11). Over one-third of girls experienced mild or stronger symptoms of anxiety and depression, whereas <15% of boys experienced symptoms of anxiety, and 20% experienced symptoms of depression. We did not find significant differences between girls and boys in terms of the impact of the sum of experienced inconveniences on anxiety. However, the effect of interaction between gender and inconveniences was close to statistical significance (*p* = 0.070). This indicates that lockdown-related anxiety should be investigated further in larger samples. In our study, we also found that the number of lockdown-related inconveniences associated with depression symptoms affected participants differently depending on their gender. In girls, the number of difficulties related to lockdown considerably impacted their symptoms of depression. This result may indicate that girls with developmental disabilities are particularly vulnerable when it comes to coping with difficulties that arise in unexpected and burdensome situations, such as the COVID-19 lockdown. The sex differences in levels of anxiety and depression occur typically in general population (10), and also in persons with intellectual disability (9). The possible explanations why the boys presented less symptoms in our study may concern social expectations regarding the male role in society, which may lead to underreporting of emotional problems (12), or variety of biological factors (10). In our study a small number of boys seemed to show increase in emotional symptoms as a function of pandemic-related inconveniences. In future studies differences between boys who do and boys who do not develop emotional symptoms should also be an object of an in-depth analysis.

The study has some limitations. First, the sample of the students is relatively small, and further studies should be conducted on larger samples of persons with developmental disabilities. Second, the baseline level of depression and anxiety symptoms before the pandemic occurred was not assessed in our study, thus, there is a possibility that there are other factors, except the pandemic-related difficulties, which influence the results. Third, the exact IQ scores for all the participants could not be obtained due to the pandemic situation. In future similar studies IQ scores should be included in regression analyses. Finally, we tested the participants only once, soon after the lockdown began. In future studies it seems important to assess emotional symptoms several times since it is possible that symptoms of depression and anxiety may relate to the amount of time spent in lockdown and may change in respect of further experienced inconveniences.

The results of our study indicate that symptoms of anxiety and depression occur frequently in persons with developmental disabilities (especially in girls). These results stand in accordance with the results of studies conducted in Ireland during the pandemic in which women also presented higher rates of anxiety and depression (13). Although in our study we were not able to verify whether the severity of the symptoms has changed compared to the period before the lockdown, the increase in the symptoms of anxiety and depression in persons with developmental disabilities should be expected in situations of school closures, limitations in trade, services, and mobility, and other troublesome restrictions. Therefore, we recommend conducting screening assessments for symptoms of anxiety and depression that are carried out similarly to those described in this paper; we also recommend the provision of psychological and psychiatric support to persons with intellectual disability and other vulnerable groups.

## Data Availability Statement

The raw data supporting the conclusions of this article will be made available by the authors, without undue reservation.

## Ethics Statement

The studies involving human participants were reviewed and approved by Ethics Committee at the Institute of Psychology, Pedagogical University of Kraków. Written informed consent to participate in this study was provided by the participants' legal guardian/next of kin.

## Author Contributions

MG and LK contributed to the concept, design of the study, and wrote the manuscript. MG organized and conducted the research. LK performed the statistical analysis and prepared the figures. All authors contributed to the revision of the manuscript and read and approved the submitted version.

## Conflict of Interest

The authors declare that the research was conducted in the absence of any commercial or financial relationships that could be construed as a potential conflict of interest.

## References

[1] Wu F Zhao S Yu B Chen Y-M Wang W Song Z-G. A new coronavirus associated with human respiratory disease in China. Nature. (2020) 580:E7. 10.1038/s41586-020-2008-3 32296181 PMC7608129

[2] Rothan HA Byrareddy SN. The epidemiology and pathogenesis of coronavirus disease (COVID-19) outbreak. J Autoimmunity. (2020) 109:1–4. 10.1016/j.jaut.2020.102433 PMC7127067 32113704

[3] Remuzzi A Remuzzi G. COVID-19 and Italy: what next? Lancet. (2020) 395:1225–8. 10.1016/S0140-6736(20)30627-9 32178769 PMC7102589

[4] World Health Organization. WHO coronavirus disease (COVID-19) dashboard. (2020). Available online at: https://covid19.who.int/region/euro/country/pl (accessed June 6, 2020).

[5] World Health Organization. WHO Director-General's opening remarks at the media briefing on COVID-19. (2020) Available online at: https://www.who.int/director-general/speeches/detail/who-director-general-s-opening-remarks-at-the-media-briefing-on-covid-19—11-march-2020 (accessed June 6, 2020).

[6] Jarynowski A Wójta-Kempa M Płatek D Czopek K. Attempt to understand public health relevant social dimensions of COVID-19 outbreak in Poland. SSRN Electron J. (2020) 4:7–44. 10.2139/ssrn.3570609

[7] Rush KS Bowman LG Eidman SL Toole LM Mortenson BP. Assessing psychopathology in individuals with developmental disabilities. Behav Modification. (2004) 28:621–37. 10.1177/0145445503259830 15296521

[8] Wallander JL Dekker MC Koot HM. Psychopathology in children and adolescents with intellectual disability: measurement, prevalence, course, and risk. In: Gidden LM editor. International Review of Research in Mental Retardation. San Diego: Academic Press. (2003). p. 93–134. 10.1016/S0074-7750(03)01003-6

[9] Glenn E Bihm EM Lammers WJ. Depression, anxiety, and relevant cognitions in persons with mental retardation. J Autism Dev Disord. (2003) 33:69–76. 10.1023/a:1022282521625 12708581

[10] Altemus M Sarvaiya N Neill Epperson C. Sex differences in anxiety and depression clinical perspectives. Front Neuroendocrinol. (2014) 35:320–30. 10.1016/j.yfrne.2014.05.004 24887405 PMC4890708

[11] Lunsky Y. Depressive symptoms in intellectual disability: does gender play a role? J Intellectual Disabil Res. (2003) 47:417–27. 10.1046/j.1365-2788.2003.00516.x 12919192

[12] Li H Morris RJ. Assessing fears and related anxieties in children and adolescents with learning disabilities or mild mental retardation. Res Dev Disabil. (2007) 28:445–57. 10.1016/j.ridd.2006.06.001 16860538

[13] Hyland P Shevlin M McBride O Murphy J Karatzias T Bentall RP. Anxiety and depression in the Republic of Ireland during the COVID-19 pandemic. Acta Psychiatr Scand. (2020) 142:249–56. 10.1111/acps.13219 32716520

[14] European Parliament. Regulation (EU) 2016/679 of the European Parliament and of the Council of 27 April 2016 on the protection of natural persons with regard to the processing of personal data and on the free movement of such data, and repealing Directive 95/46/EC. EUR-Lex. (2016). Available online at: https://eur-lex.europa.eu/eli/reg/2016/679/oj (accessed June 6, 2020).

[15] World Health Organization. ICD-10 guide for mental retardation. Geneva (1996). Available online at: https://apps.who.int/iris/handle/10665/63000 (accessed December 18, 2020).

[16] Spitzer RL Kroenke K Williams JBW Löwe B. A brief measure for assessing generalized anxiety disorder. Archi Internal Med. (2006) 166:1092. 10.1001/archinte.166.10.1092 16717171

[17] PHQ. Patient Health Questionnaire (PHQ) Screeners. Available online at: https://www.phqscreeners.com/ (accessed June 6, 2020).

[18] Mapi Research Trust. Covid-19 Mapi reseach trust update and guidance. Available online at: https://mapi-trust.org (Udostepniono czerwiec 6, 2020).

[19] Acquadro C Conway K Giroudet C Mear I. Linguistic validation manual for health outcome assessments. Lyon: Mapi Research Trust (2012).

[20] Wild D Grove A Martin M Eremenco S McElroy S Verjee-Lorenz A. Principles of good practice for the translation and cultural adaptation process for patient-reported outcomes (PRO) measures: report of the ISPOR task force for translation and cultural adaptation. Value Health. (2005) 8:94–104. 10.1111/j.1524-4733.2005.04054.x 15804318

[21] Kroenke K Spitzer RL Williams JB. The PHQ-9: validity of a brief depression severity measure. J Gen Internal Med. (2001) 16:606–13. 10.1046/j.1525-1497.2001.016009606.x 11556941 PMC1495268

[22] Razykov I Ziegelstein RC Whooley MA Thombs BD. The PHQ-9 versus the PHQ-8 — Is item 9 useful for assessing suicide risk in coronary artery disease patients? Data from the Heart and Soul Study. J Psychosomatic Res. (2012) 73:163–8. 10.1016/j.jpsychores.2012.06.001 22850254

[23] Kroenke K Strine TW Spitzer RL Williams JBW Berry JT Mokdad AH. The PHQ-8 as a measure of current depression in the general population. J Affective Disord. (2009) 114:163–73. 10.1016/j.jad.2008.06.026 18752852

[24] The jamovi project. jamovi. (Version 1.2). [Computer Software]. (2020). Available online at: https://www.jamovi.org (accessed June 6, 2020).

[25] R Core Team. R: A Language and environment for statistical computing. (Version 3.6). [Computer software]. (2019). Available online at: https://www.r-project.org/ (accessed June 6, 2020)

[26] Gallucci M. General analyses for linear models. (Version 2.0.5). [jamovi module]. (2019). Available online at: https://gamlj.github.io (accessed June 6, 2020).

[27] Lüdecke D. sjPlot: Data visualization for statistics in social science. (Version 2.8.4). [R package]. (2020) Available online at: https://cran.r-project.org/package=sjPlot (accessed June 6, 2020).

